# Psychedelics meet human brain organoids: insights into proteomics and potential for Alzheimer’s disease treatment

**DOI:** 10.3389/frdem.2025.1605051

**Published:** 2025-08-04

**Authors:** Xenia Androni, Rachel J. Boyd, Paul B. Rosenberg, Vasiliki Mahairaki

**Affiliations:** ^1^Department of Genetic Medicine, Johns Hopkins School of Medicine, Baltimore, MD, United States; ^2^Division of Geriatric Medicine and Gerontology, Johns Hopkins School of Medicine, Baltimore, MD, United States; ^3^The Richman Family Precision Medicine Center of Excellence in Alzheimer’s Disease, Johns Hopkins School of Medicine, Baltimore, MD, United States; ^4^Department of Psychiatry and Behavioral Sciences, Johns Hopkins School of Medicine and Johns Hopkins Bayview Medical Center, Baltimore, MD, United States

**Keywords:** Alzheimer’s disease, brain organoids, psychedelics, stem cells, neuroplasticity

## Abstract

Alzheimer’s disease (AD) is characterized by a long preclinical phase lasting more than a decade before the onset of its clinical phase of mild cognitive impairment (MCI) or dementia. Recent advances in psychedelic research underscore numerous neuroplastogenic and anti-inflammatory alterations induced by these compounds, making them promising therapeutic candidates for AD. In this mini review, we will briefly summarize the existing literature using human cerebral organoids to study the molecular and metabolic changes caused by various psychedelic compounds, focusing on their potential therapeutic applications for AD.

## 1 Introduction

Alzheimer’s disease (AD) is characterized by a long preclinical phase lasting more than a decade before the onset of its clinical phase of mild cognitive impairment (MCI) or dementia (Sperling et al., 2011; Bateman et al., 2012; Dubois et al., 2016). Its pathogenesis involves a cascade of interconnected biochemical and cellular changes, including the accumulation of beta-amyloid (Aβ) fibrils in extracellular plaques and the hyperphosphorylation of Tau in intracellular tangles. These alterations lead to neuroinflammation, synaptic dysfunction, neuronal degeneration, and ultimately to cognitive and functional decline (De Strooper and Karran, 2016). Although genetics plays a significant role (Sims et al., 2020), the precise causes and sequence of these pathological events are not yet completely understood, which can potentially explain the recurring therapeutic failures in AD research. Since treatments may be most effective in the early stages of the disease, it is crucial to identify AD in presymptomatic individuals and those experiencing cognitive decline before reaching the clinically defined MCI or dementia.

In the hippocampus, prefrontal cortex, and other areas of the brain that are susceptible to AD pathology, 5-HT2A receptors are highly present (Bryson et al., 2017). Psychedelic drugs, including d-lysergic acid diethylamide (LSD), psilocybin, 5-methoxy-N,N-dimethyltryptamine (5-MeO-DMT), and DMT are potent serotonergic agonists with affinity to 5-hydroxytryptamine receptors (5-HTRs) (Nichols, 2016). Recent advances in psychedelic research underscore the numerous connections between these compounds and cognitive/affective alterations observed in older adults (Aday et al., 2020), making them appealing therapeutic candidates for AD.

Studies in rodents suggest that psychedelics support neurogenesis, neuroplasticity, and neuronal maturation by enhancing the development of neurites, dendritic spines, and synapses in neural progenitor cells, particularly where 5-HT2A receptors are highly expressed (Lima da Cruz et al., 2018; Morales-Garcia et al., 2020; Ly et al., 2018). These studies have revealed many cellular and molecular mechanisms of these drugs; however, expanding these findings to a human-relevant model is critical for evaluating the therapeutic potential of psychedelics.

Given the many differences between human and rodent brains (Xu et al., 2022), it has been challenging to model human brain physiology (particularly that of the hippocampus) in the laboratory. Laboratory-synthesized, human-derived 3D brain organoids represent an *in vitro* system that effectively models many aspects of the molecular architecture of the human brain (Chen et al., 2019; Porci’uncula et al., 2021). In this mini review we will briefly summarize the existing literature using cerebral organoids to study the molecular and metabolic alterations caused by various psychedelic compounds, focusing on their potential therapeutic applications for AD.

## 2 5-MeO-DMT effects

5-MeO-DMT is a short-acting psychedelic tryptamine that acts as a serotonin receptor agonist with affinity for other receptors, as well as serotonin and norepinephrine transporters (Ermakova et al., 2022).

5-MeO-DMT has been shown to induce anti-inflammatory pathways and other proteomic alterations in 45-day old human embryonic stem cell-derived cerebral organoids. Following exposure to 5-MeO-DMT for 24-h, shot-gun mass spectrometry (MS) revealed widespread changes in protein expression within toll-like receptor- and Gq-coupled receptor-mediated signaling cascades, ultimately leading to downregulation of transcriptional regulators of inflammatory cytokines, NFAT and NF-*κβ* (Dakic et al., 2017). Other downregulated proteins included srGAP, which is critical for the processes underpinning synaptic plasticity and higher cognitive functions (Dakic et al., 2017; Nguyen Thi Thanh et al., 2018), and mGluR5, which contributes to the rewarding effects of various drugs of abuse; thus, supporting the hypothesis that psychedelics carry a low risk of addiction (Johnson et al., 2018).

**Figure 1:**
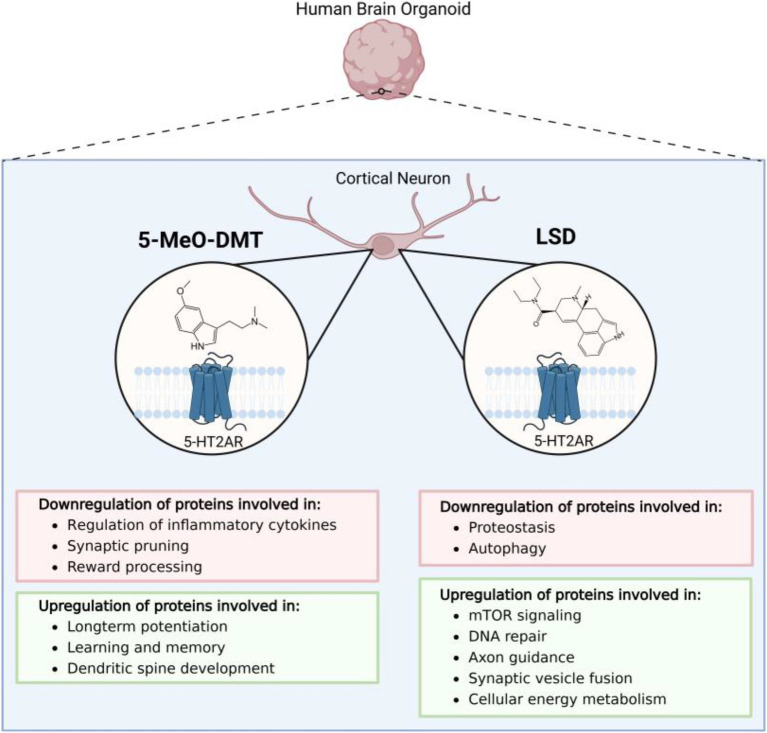
An overview of the state of the current literature evaluating the effects of psychedelic compounds in human cell-derived 3D brain organoids. Presently, only proteomics studies evaluating the effects of d-lysergic acid diethylamide (LSD) and 5-methoxy-N,N-dimethyltryptamine (5-MeO-DMT) have been applied to cerebral organoid models.

5-MeO-DMT treatment upregulated the expression of NMDAR, CaMK2, and CREB—signaling molecules involved in long-term potentiation, learning, and memory (Dakic et al., 2017; Caya-Bissonnette and B’eique, 2024). 5-MeO-DMT significantly increased EFNB2, EPHB, intersectin, ELMO1, CDC42, RAC1, and integrins, which promote dendritic spine development. Finally, 5-MeO-DMT was shown to suppress cell death-related pathways upon activation of *σ*1-RS, which induces neuroprotection by modulating intracellular calcium levels and inhibiting the expression of pro-apoptotic genes (Figure 1) (Mueller et al., 2013).

## 3 LSD effects

LSD is another psychedelic that predominantly exerts its effects through 5-HT2A receptor antagonism, but also binds to dopaminergic, adrenergic, and other serotonergic receptors (De Gregorio et al., 2021). Furthermore, LSD allosterically binds tropomyosin receptor kinase B (TrkB), promoting its interaction with brain-derived neurotrophic factor (BDNF); a key molecule that mediates plastic changes underlying learning and memory (Figure 1) (Moliner et al., 2023).

The impact of LSD on neural plasticity-related pathways has been evaluated using 45-day-old human-induced pluripotent stem cell (hiPSC)-derived cerebral organoids (Ornelas et al., 2022). After treatment with 10 nM LSD for 24-h, liquid-chromatography (LC)-MS revealed proteomic alterations involved in common cellular processes including DNA replication, mTOR signaling cascades, and dopamine neurotransmitter release cycle. Specifically, mTOR was significantly upregulated, which may promote psychedelic-induced structural plasticity in the prefrontal cortex (Ly et al., 2018). Other affected pathways reflect neuroplasticity and synaptic reorganization processes, particularly axon guidance, synaptic vesicle cycle, and long-term depression (Ornelas et al., 2022). In fact, there is evidence that neuronal plasticity is stimulated by LSD through both 5-HT2A and mTOR signaling (Ly et al., 2018).

In a subsequent study (Costa et al., 2024), hiPSC-derived 45-day old human cortical organoids were then exposed to 100 nM LSD for 24 h. LC/MS-MS-based shotgun proteomic analysis revealed a significant shift in the abundance of multiple proteins that modify processes involved in proteostasis, energy metabolism, and neuroplasticity. Most proteostasis proteins were downregulated, possibly prolonging the lifespan of synaptic proteins by slowing turnover rates, although it is unclear whether LSD regulates proteostasis directly or through indirect homeostatic effects. In addition, LSD altered the abundance of proteins associated with glycolysis, the TCA cycle and oxidative phosphorylation, and also increased lactate production, implying that psychedelics could trigger metabolic alterations to meet the high demands of neuroplasticity (Watts et al., 2018). LSD exposure led to upregulation of synaptic vesicle fusion proteins, suggesting an effect on neuroplasticity and neurotransmitter release.

## 4 Discussion

Preliminary research has highlighted the antidepressant, anxiolytic, and anti-addictive features of classic psychedelics (Watts et al., 2018). Furthermore, preclinical and neuroimaging studies point to a variety of biological mechanisms of action of psychedelics, including structural and functional enhancement of neuroplasticity (Lima da Cruz et al., 2018; Ly et al., 2018), anti-inflammatory properties (Flanagan and Nichols, 2018), shifts in critical signaling pathways (i.e., BDNF) (Ly et al., 2018; Hutten et al., 2020), and modifications of functional neural connectivity (Carhart-Harris et al., 2017; Barrett et al., 2020; Preller et al., 2020). The key pathophysiological processes of AD include decreased functional brain activity and connectivity (Dennis and Thompson, 2014), reduced serotonergic neurotransmission (Smith et al., 2017; Mecca, 2019) associated with neuropsychiatric symptoms (Butzlaff and Ponimaskin, 2016; Chakraborty et al., 2019), neuroinflammation, (Kinney et al., 2018) and alteration of key signaling pathways (i.e., BDNF) (Peng et al., 2005; Tanila, 2017). Therefore, for many patients with AD, classic psychedelics may offer therapeutic advantages that merit further investigation.

Although two-dimensional (2D) iPSC-derived neuronal cultures are valuable tools to simulate cellular responses, only one study has focused on the neuroprotective impact of the endogenous hallucinogen N,N-dimethyltryptamine (DMT) on human cortical neurons derived from iPSCs, monocyte-derived macrophages, and dendritic cells (Szabo et al., 2016). Studies investigating the effects of psychedelics in iPSC-derived neurons are exceedingly rare and, to our knowledge, have not yet been conducted in AD patient-derived models. Furthermore, we are not aware of any data that exist from post-mortem AD brain tissue studies. In light of these gaps, iPSC-derived organoids offer a valuable, sophisticated system that closely mimics the spatial architecture of the human brain and incorporates complex cell-to-cell interactions, which may influence drug responses (Salerno and Rehen, 2024).

Human brain organoids provide a unique *in vitro* imitation of physiologically relevant complex functions and processes of the human brain (Logan et al., 2019; Whiteley et al., 2022), and thus provide valuable data connecting preclinical and clinical studies. Importantly, organoids can be grown using patient-derived iPSCs to serve as tools for precision medicine approaches. Given the highly psychoactive properties of psychedelic substances and the heterogeneous neurobehavioral reactions they evoke (Moujaes et al., 2023), patient-derived organoids can be used to evaluate personalized treatment strategies that achieve optimal efficacy with minimal adverse effects (Park and Mook-Jung, 2022).

Considering that serotonergic degeneration is observed early in the course of AD (Smith et al., 2023) and that psychedelic compounds mediate their effects primarily through the 5-HT2A receptor, hindbrain serotonergic organoids could offer a unique platform to investigate how psychedelics influence serotonergic pathways in the context of AD pathology (Valiulahi et al., 2021; Zivko et al., 2024). The methodological framework developed for studying the response of serotonergic hindbrain organoids to escitalopram (Zivko et al., 2024), a selective serotonin reuptake inhibitor (SSRI), suggests that this platform can be used to explore the potential of psychedelics to treat AD.

There is growing evidence that late-life depressive symptoms are associated with an increased risk of incident dementia, a concept that has been codified into the construct of ‘mild behavioral impairment’ (Creese and Ismail, 2022). In the data set of the National Alzheimer’s Coordinating Center, the majority of participants who progressed from normal to impaired cognition presented behavioral symptoms prior to cognitive changes (Wise et al., 2019). Thus, the mechanisms underlying late-life depression may also be the basis for neurodegenerative disease. Many of the mechanisms identified by the aforementioned organoid studies are common to depression and AD, including decreased serotonergic innervation, neuroinflammation, and disruptions in crucial signaling pathways, including BDNF (Mendes-Silva et al., 2016; Linnemann and Lang, 2020; Bajaj and Mahesh, 2024). Clinical trials have shown that a single dose of a psychedelic can induce lasting physiological changes across multiple neural pathways without the need for sustained or repeated exposure to maintain these effects (Knudsen, 2023). This is analogous to the observation that a single dose of psilocybin was effective in treating major depression both in the short term (3 weeks) (Goodwin et al., 2022) and in a long-term open-label follow-up over 52 weeks (Goodwin et al., 2025), suggesting that a single psychedelic dose can induce long-term alterations in the human brain.

Psilocybin is another psychedelic that is being evaluated as a treatment for several neurological disorders (Carhart-Harris et al., 2021; Schindler et al., 2021; Daws et al., 2022). Although its application as a medical intervention for AD has been understudied, it has been recognized as a breakthrough therapy for major depressive disorder (Raison et al., 2023; Zheng et al., 2024). Considering the potential of psilocybin to induce neurogenesis, synaptogenesis, and synaptic plasticity (Jefsen et al., 2020; Shao et al., 2021; Raval et al., 2021), it may represent a strong candidate for trials in human brain organoids, with the aim of exploring its potential therapeutic benefit for AD. Furthermore, depression and anxiety are prominent symptoms of AD and can accelerate the progression of the disease (Ma, 2020; Agüera-Ortiz et al., 2021). Therefore, psilocybin may alleviate affective symptoms and even delay the course of the disease.

Despite a growing body of evidence supporting the therapeutic potential of psychedelics across various medical conditions, their use remains controversial due to lingering stigma around historical misuse. Notably, several perceived risks, such as addiction and neurotoxicity, have been refuted by recent research (Schlag et al., 2022). However, other potential risks, such as the exacerbation of delusions or hallucinations following high-dose psychedelic administration, remain a concern, particularly in individuals with advanced AD (Scarmeas et al., 2005). As a result, research into the therapeutic potential of psychedelics is increasingly focused on the earliest stages of the disease (Garcia-Romeu et al., 2022). In this context, a rational approach may involve eliminating the hallucinogenic effects of these compounds while preserving their therapeutic benefits, potentially minimizing adverse side effects (Yin and Gao, 2023). Moreover, to support this effort, advanced model systems such as organoids could offer the potential to understand the mechanisms underlying the heterogeneity of the clinical response, guiding the development of more targeted therapeutic strategies (Zhou et al., 2023; Giorgi et al., 2024; Smirnova and Hartung, 2024).

Altogether, strong evidence suggests that psychedelic drugs mediate plastogenic and anti-inflammatory processes in brain regions involved in AD pathology, which makes them promising cognitive enhancers and prospective therapeutic candidates. Given the high value of human cerebral organoids as tools for conducting preclinical research in a human-relevant environment, more studies in this direction are required to gain a comprehensive understanding of the mechanisms behind the neurorestorative impact of these compounds on the human brain.
